# The effect of altruistic tendency on fairness in third-party punishment

**DOI:** 10.3389/fpsyg.2015.00820

**Published:** 2015-07-02

**Authors:** Lu Sun, Peishan Tan, You Cheng, Jingwei Chen, Chen Qu

**Affiliations:** ^1^Center for Studies of Psychological Application, School of Psychology, South China Normal UniversityGuangzhou, China; ^2^Primary School Affiliated to South China Normal UniversityGuangzhou, China; ^3^Department of Psychological and Brain Sciences, Dartmouth CollegeHanover, NH, USA; ^4^School of Economics and Management and Scientific Laboratory of Economics Behaviors, South China Normal UniversityGuangzhou, China

**Keywords:** altruistic tendency, third-party punishment, unfair, ERP, MFN

## Abstract

Third-party punishment, as an altruistic behavior, was found to relate to inequity aversion in previous research. Previous researchers have found that altruistic tendencies, as an individual difference, can affect resource division. Here, using the event-related potential (ERP) technique and a third-party punishment of dictator game paradigm, we explored third-party punishments in high and low altruists and recorded their EEG data. Behavioral results showed high altruists (vs. low altruists) were more likely to punish the dictators in unfair offers. ERP results revealed that patterns of medial frontal negativity (MFN) were modulated by unfairness. For high altruists, high unfair offers (90:10) elicited a larger MFN than medium unfair offers (70:30) and fair offers (50:50). By contrast, for low altruists, fair offers elicited larger MFN while high unfair offers caused the minimal MFN. It is suggested that the altruistic tendency effect influences fairness consideration in the early stage of evaluation. Moreover, the results provide further neuroscience evidence for inequity aversion.

## Introduction

Altruistic punishment refers to punishment imposed by individuals who punish free riders in the group although it is costly and yields no material benefits for the punishers. This punishment may achieve and sustain social cooperation (Fehr and Gächter, 2002). Altruistic punishment includes second-party punishment and third-party punishment. Second-party punishment refers to punishment inflicted by the person who suffered from the violation. For example, in the ultimatum game (Güth et al., 1982), the receiver can reject the unfair offer from the proposer; the rejection of the receiver is regarded as second-party punishment. Third-party punishment refers to the circumstances in which a third party who did not directly suffer from the violation is willing to pay a cost to punish the violator (Fehr and Fischbacher, 2004a,b).

The third-party punishment of dictator game is an effective tool to explore altruistic punishment and fairness distribution (Eckel and Grossman, 1996). However, the costly punishment violates the classic homo economicus theory that humans are always in pursuit of profit maximization. In the game, the dictator can decide how to distribute the money while the receiver can only accept unconditionally. After observing the distribution and level of cooperation, the otherwise disinterested third party can determine whether to pay a cost to punish the individuals who violate the cooperation social norms (Fehr and Fischbacher, 2003). Compared with second-party punishment, third-party punishment could minimize violations of social norms and maintain social equality; examples of third-party punishers are the justice system and police (Marlowe et al., 2008).

Inequality aversion theory holds that third-party punishment is the result of someone resisting inequality. More specifically, individuals abandon self-interest voluntarily to pursue a more equitable result. Several studies have found that participants exhibit aversion against inequality and impose punitive measures to reduce the pecuniary gap between people (Fehr and Schmidt, 1999; Dirk and Martin, 2006). Bolton and Ockenfels (2000) found that punishment is imposed in order to make the violator’s amount of money close to the average level.

Several lines of evidence support the viewpoint of inequity aversion theory. First, Falk and Fischbacher’s (2006) reciprocity theory predicts that people tend to reward kind actions and punish unkind actions. Their evidence suggests that the evaluation of a kind action is based not only on its consequences but also on its underlying intention.

Second, some researchers explain third-party punishment in terms of cognition and emotion. When people internalize a specific culture, morality that follows social norms will be formed. Internal self-punishment will be elicited if the social norms are violated (Masclet et al., 2003). Masclet et al. (2003) believes the internal pressure is a kind of sense of guilt, which is the reason why people want to punish violators even when they have not experienced directly. When observing violations committed by others, people can experience negative emotions such as the desire for revenge, the urge to fight or anger (Bosman and van Winden, 1999; Glaeser and Sacerdote, 2000; Decker et al., 2003; Abbink et al., 2004).

Neuroscience research provides further evidence for inequity-aversion theory (Pérez and Kiss, 2012). In one functional magnetic resonance imaging (fMRI) study (Singer et al., 2006), male volunteers who observed an unfair confederate receiving pain showed lower empathy-related activation, accompanied by reward-related activation that might represent the desire for revenge. A positron emission tomography (PET) study (De Quervain et al., 2004) investigating the neural mechanism of third-party punishment in a trust game found that subjects with stronger activations in the dorsal striatum, which has been implicated in the processing of rewards, were willing to incur greater costs in order to punish. Moreover, when subjects, acting as third parties, determined to pay a cost to punish the norm violators, ventral medial prefrontal cortex (vmPFC) and medial prefrontal cortex (mPFC) showed greater activation than in costless punishment. It is thought that there is a trade-off relation underling the monetary punishment behavior. Specifically, participants need to weigh the emotional satisfaction and monetary loss from the punishment at the same time, which is indicated by vmPFC and mPFC, to integrate the cognitive conflict with the decision-making processing.

Actually, a number of people do not enact third-party punishment. This is not inconsistent with inequity aversion theory, which holds that attitudes on inequity distribution should affect third-party punishment. However, aside from the research on the neural mechanisms of third-party punishment, only a few studies have explored individual differences in this behavior. Haruno and Frith (2009) explored how social value orientation, as an individual difference, affects anchoring attitudes toward resource division. Results revealed that the prosocials disliked large absolute differences in distributions (inequity aversion), whereas the individualists were unaffected by such differences. Moreover, the extent of inequity aversion in prosocials was predicted by activity in the amygdala and appeared to be impervious to cognitive load.

In the current study, altruistic tendency was introduced as an individual difference predicting altruistic punishment. In addition to behavioral results from the punishment of dictator game, event-related potential (ERP) technique was employed to assess the neural process of fairness consideration in altruistic punishments.

Medial frontal negativity (MFN) was referred to a family of negative-going ERPs peaking between 200 and 350 ms at frontocentral recording sites. MFN is associated with performance evaluation, including error-related negativity (ERN; Falkenstein et al., 1990; Gehring et al., 1990) and feedback-related negativity (FRN; Miltner et al., 1997). Some studies have found that MFN is sensitive to the violation of social expectancy or social norms (Boksem and De Cremer, 2010; Van der Veen and Sahibdin, 2011; Wu et al., 2011, 2012; Qu et al., 2013b). In an ultimatum game study, Wu et al. (2011) reported that compared with moderately unequal offers, highly unequal offers generated larger MFN indicating that the MFN can reflect a general violation of social expectancy. In present study, we predict that high altruists and low altruists would show different patterns in MFN because of different social expectancy.

We also examined another ERP component, P300, as an ERP component that has attracted interest in emotion and attention research. As shown in previous studies, P300 is sensitive to the valence and the magnitude; positive feedback generated larger P300 negative feedback (Wu and Zhou, 2009; Leng and Zhou, 2010; Qu et al., 2014). Yeung and Sanfey (2004) posited that the P300 is modulated by the individual’s attention and emotional experience in result evaluations. Researchers have also found that P300 is significantly larger in reward conditions than in punishment or non-reward conditions (Hajcak et al., 2005; Bellebaum and Daum, 2008).

Therefore, the present research, employing the ERP technique, tested whether and how altruistic tendency affects third-party punishment. For behavioral results, we hypothesized that unfair offers would elicit more third-party punishments according to inequity aversion theory, and altruistic tendencies would moderate third-party punishments. Compared with low altruists, high altruists would show more third-party punishment when observing the unfair offer. As for the ERP results, MFN outcomes with larger violation of expectancy should elicit larger MFN than outcomes in line with expectancy. For high altruists, an expected outcome is the fair offer, while the unfair offer is an unexpected result. The opposite pattern should be found for low altruists. Therefore, we expected that when the high altruists observed the unfair offer, greater MFN would be elicited, whereas the low altruists would show greater MFN when a fair offer was observed. Considering the P300 is associated with the emotional arousal, we assume that the unfair offer would elicit larger P300 than the fair offer. High and low altruists will show variation in the pattern of P300, and show different punishment behavior.

## Materials and Methods

### Participants

Seventy right-handed undergraduate students from the South China Normal University voluntarily participated in the first stage of the research, and then a distribution task was used as a pretest through which thirty-two participants (22 females and 10 males,18–24 years of age) were selected to take part in the formal experiment. The mean age of the participants was 21.4 years. Participants reported no physical or mental illness and reported normal eyesight. Informed consent was obtained from all participants, and the research was approved by the Human Research Ethics Committee of South China Normal University.

### Material

The third-party punishment dictator game was presented using the E-Prime experimental program. We applied color bars to present the allocations of the dictator. The horizontal viewing angle of each target picture was 3° and the vertical viewing angle was 1.5°.

### Design and Procedure

The design was a two factor mixed design with the first factor referring to the level of fairness (Fair offer, Medium Unfair offer, High Unfair offer) and the second factor referring to the altruistic tendency (High, Low). Recent studies have shown that the dictator game is an effective paradigm to differentiate altruistic behavior (Benenson et al., 2007; Carpenter et al., 2008; Rotemberg, 2008). Thus we used the dictator game to identify participants with high and low altruistic tendencies. Participants were presented with a pair of rewards (totally 100 Yuan) for self and the other, 35 times. Three predetermined allocations including a low altruistic tendency allocation (90:10), medium altruistic tendency allocation (70:30), and high altruistic tendency allocation (50:50) were presented. We assigned participants to a certain category (high altruist, low altruist) if they made consistent decisions more than 75% of the time. Finally, 32 participants were selected for the formal experiment, 16 (3 male, 13 female) high altruists and 16 (7 males, 9 females) low altruists.

Our experiment adopted a modified paradigm of the third-party punishment of dictator game (Qu et al., 2014). In the formal task, participants were assigned a role of third party and received an initial endowment of 50 Yuan. They first witnessed a distribution of 100 Yuan between two players (the dictator and the receiver). Subsequently, the participants would have an opportunity to adjust the distribution by subtracting 15 Yuan from their endowment to turn the unfair offer into a fair offer in order to punish the dictator. The fairness factor includes three levels: Fair offer refers to both dictator and receiver owning 50 Yuan, Medium Unfair offer refers to 70 Yuan for the dictator and 30 Yuan for the receiver, and High Unfair offer refers to 90 Yuan for the dictator and 10 Yuan for the receiver. In other words, if the distribution is 90:10, the participant can spend 15 Yuan to punish the dictator, thus the distribution result will become 50:50. Before starting the dictator game, the participants were informed that the dictator results come from another group of over 300 participants who had participated previously. All participants were paid 20 Yuan as a basic payment, and were informed that an extra reward would be paid according to their decisions in the task. We randomly chose one trial’s balance as his extra reward. After the experiment, we asked all the participants whether they believe that they were playing against the real human players, most of the participants considered they encountered the real person in the game. Participants were debriefed, paid, and thanked.

**FIGURE 1:**
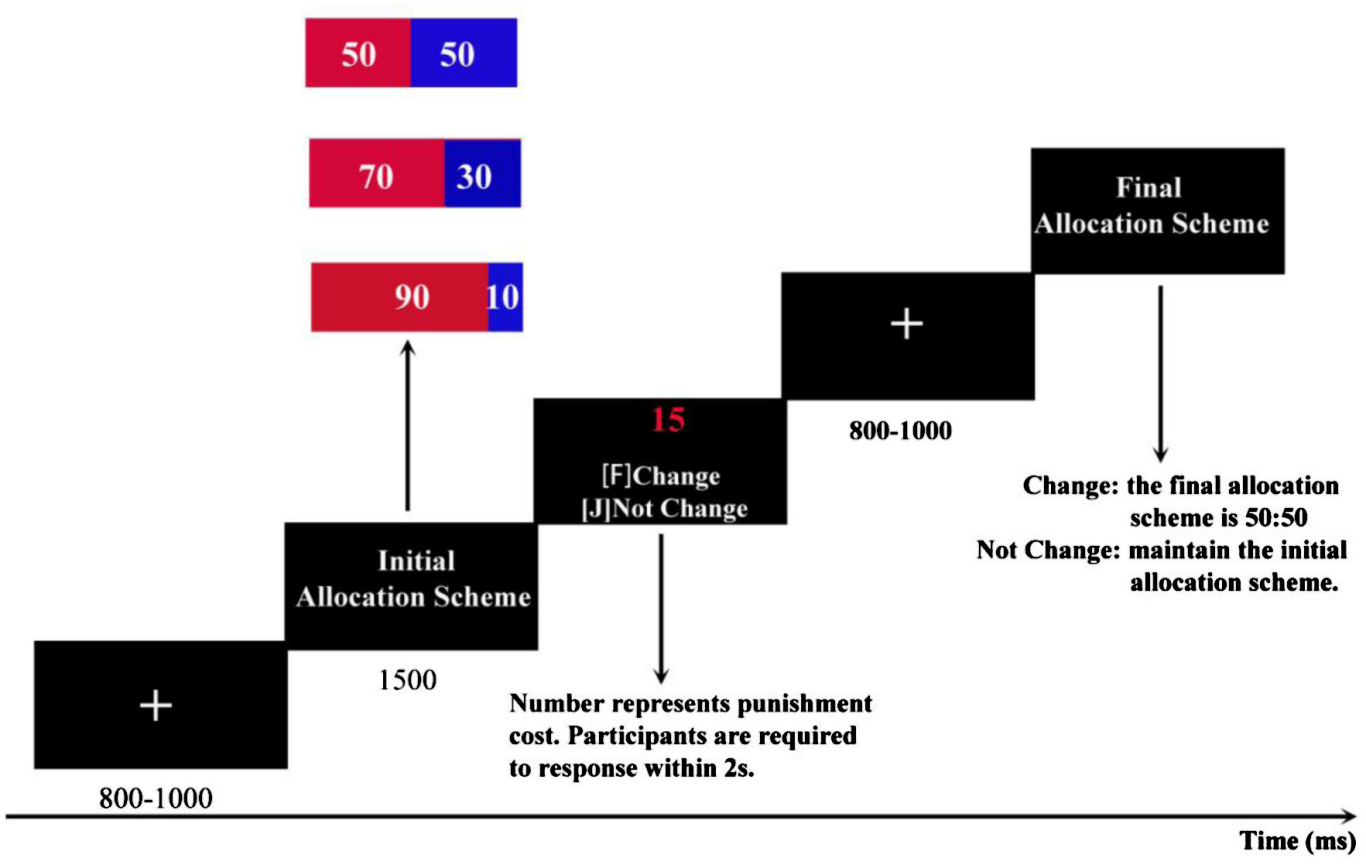
**Sequence of events in a single trial of the third-party punishment event-related potential (ERP) study.** ERP time locked to initial allocation scheme.

Participants were seated comfortably in an audio-shielded room with a fabric cap and were required to gaze at the screen center, which was 1 m away in front of their eyes. Participants were asked to read detailed task instructions. All participants had 20 trials to practice until they fully understood the task. The formal experiment process is shown in **Figure 1**. In each round of the game, every participant was required to gaze at a fixation point that appeared as “+” in the center of the screen for 800–1000 ms. Then the initial allocation scheme of the dictator was shown on the screen. A color bar was presented for 1500 ms, with a portion in red on the left side representing the score of the dictator, and a portion in blue on the right side representing the score of the recipient. Next, a selection window was given, and the participant was prompted to press the “F” or “J” key on the keyboard within 2 s to indicate whether to change the allocation of the dictator. After the decision-making, the subject would see a fixation point “+” for 800–1000 ms and then observe the final distribution and his/her score in this round. This was one trial of the task. If the participant was willing to turn the unfair offer into a fair offer, he/she would be deducted 15 Yuan; if not, the final offer would be consistent with the initial allocation, and thus the third party (participant) would retain 50 Yuan. The three fairness levels were presented in random order and each condition had 50 trials, thus there were 150 trials in the experiment. From the beginning of the formal task, EEG data and the frequency of punishment by each participant were recorded.

### Record of ERP

EEGs were recorded from 32 scalp sites at 500 Hz rate. All electrodes were embedded in an elastic cap. The EEG signals were amplified with a band pass of 0.01–100 Hz by online filtering of BrainAmps (Brain Products, Munich). All electrode recordings were referenced online to the right mastoid and off-line re-referenced to the average of the left and right mastoids. The horizontal electrooculograms (HEOGs) were monitored with off-line electrodes located in both laterals of the eyes, the vertical electrooculograms (VEOGs) were placed above and below the left eye. Brain Vision Analyzer (analysis software) was performed to exclude the eye-movement signal by using independent component analysis for continuous data. Trials with EEG artifacts that exceeded ±80 μV from the mean amplitude during the recording epoch were eliminated. EEG data were measured and analyzed by no-phase-shift low-pass digital filtering of 20 Hz. EEG epochs of 1000 ms, within a 200 ms pre-stimulus baseline, would be superimposed to analyze after the initial allocation scheme.

Based on the procedure used in previous research (Gering and Willoughby, 2002; Hajcak et al., 2005), MFN is maximal on the frontocentral electrodes, thus data from electrode sites Fz, FCz, Cz were pooled for analysis. For P300 analysis, the largest amplitude appears in the posterior sites, so a pooling of Cz, Pz electrodes was used for analysis. The mean amplitude of MFN is between 290 and 390 ms, while the mean amplitude of P300 is between 400 and 600 ms.

## Results

### Behavioral Results

The frequency of the third-party punishment by level of high and low altruistic tendency was presented in **Table 1**. Considering the possible gender difference, we regard the gender factor as a covariate. A 3 (fairness: Fair, Medium Unfair, High Unfair) × 2 (altruistic tendency: High, Low) repeated measures analysis of variance (rm-ANOVA) was applied to analyze the frequency of third-party punishment. Altruistic tendency was the between-subjects variable, fairness was the within-subjects variable, gender was covariate. The results show significant main effects of the fairness [*F*(2,58) = 22.542, *p* < 0.001] and altruistic tendency [*F*(1,29) = 12.505, *p* = 0.001]. The interaction between fairness and altruistic tendency was significant, [*F*(2,58) = 5.352, *p* = 0.006]. Furthermore, the simple effect analysis suggested that for low altruists, the medium and high unfair offers generated more punitive behaviors than fair offers (both *p* < 0.001), the difference of the punishments between fair and medium unfair offers was significant, *p* = 0.002. For high altruists, they significantly showed less punishments in fair and medium unfair offers than in high unfair offers (both *p* < 0.001), but the difference between fair and medium unfair offers was not significant.

**Table 1:** Frequency of third-party punishment by level of altruistic tendency.

Allocation scheme	High altruists	Low altruists
50: 50	0	0
70: 30	14.19 ± 20.50	5.12 ± 5.30
90: 10	44.88 ± 6.75	27.81 ± 10.76

### ERP Results

**FIGURE 2:**
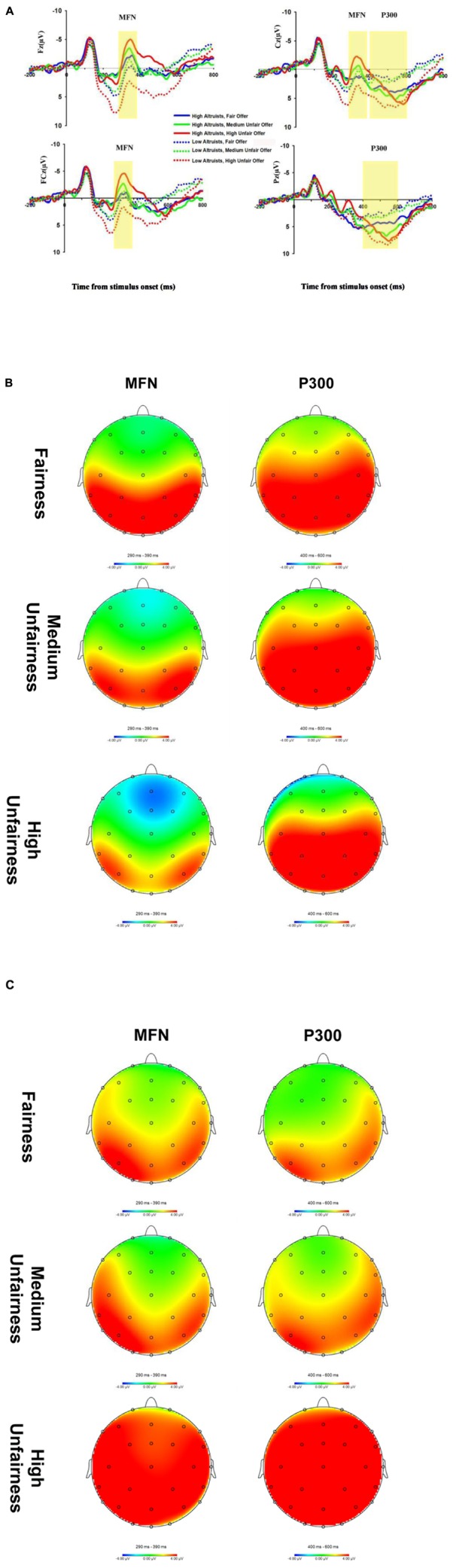
**Event-related potential data associated with high and low altruists. (A)** Grand-average ERP waveforms to different allocations for high altruists and low altruists. The shaded 290–390 ms and 400–600 ms time windows were used to measure the medial frontal negativity (MFN) and P300 magnitude, respectively. **(B)** The scalp distribution of the MFN and P300 difference waves on different levels of unfairness of the high altruists. **(C)** The scalp distribution of the MFN and P300 difference waves on different levels of unfairness of the low altruists.

Two female participants were excluded because of displaying excessive artifacts in EEG recording. The remaining 30 participants included 15 high altruists (3 male and 12 female) and 15 low altruists (7 male and 8 female). **Table 2** presents the means and SD of MFN and P300 amplitudes in three different distribution schemes. **Figure 2** shows the average waveforms to different allocations for high and low altruists.

**Table 2:** Average amplitude and SD of medial frontal negativity (MFN) and P300 in different distribution schemes, by level of altruistic tendency.

Electrode	Fair (50: 50)				Medium unfair (70: 30)				High unfair (90: 10)			
	High altruists		Low altruists		High altruists		Low altruists		High altruists		Low altruists	
	*M*	SD	*M*	SD	*M*	SD	*M*	SD	*M*	SD	*M*	SD
**MFN**					
Fz	-1.15	4.79	0.93	4.14	-2.03	4.37	0.17	3.11	-3.31	3.67	3.65	4.07
FCz	-0.27	3.65	0.76	3.91	-1.25	4.18	0.27	2.89	-3.15	3.44	2.93	3.77
Cz	1.57	4.04	1.69	4.20	0.48	3.99	1.45	3.13	-1.12	2.91	3.91	3.95
**P300**					
Cz	3.32	4.84	1.08	4.41	4.23	2.96	1.38	3.26	4.43	3.55	5.46	5.02
Pz	4.02	3.99	2.18	3.48	5.62	3.66	2.64	3.31	6.26	4.19	7.24	4.43

**FIGURE 3:**
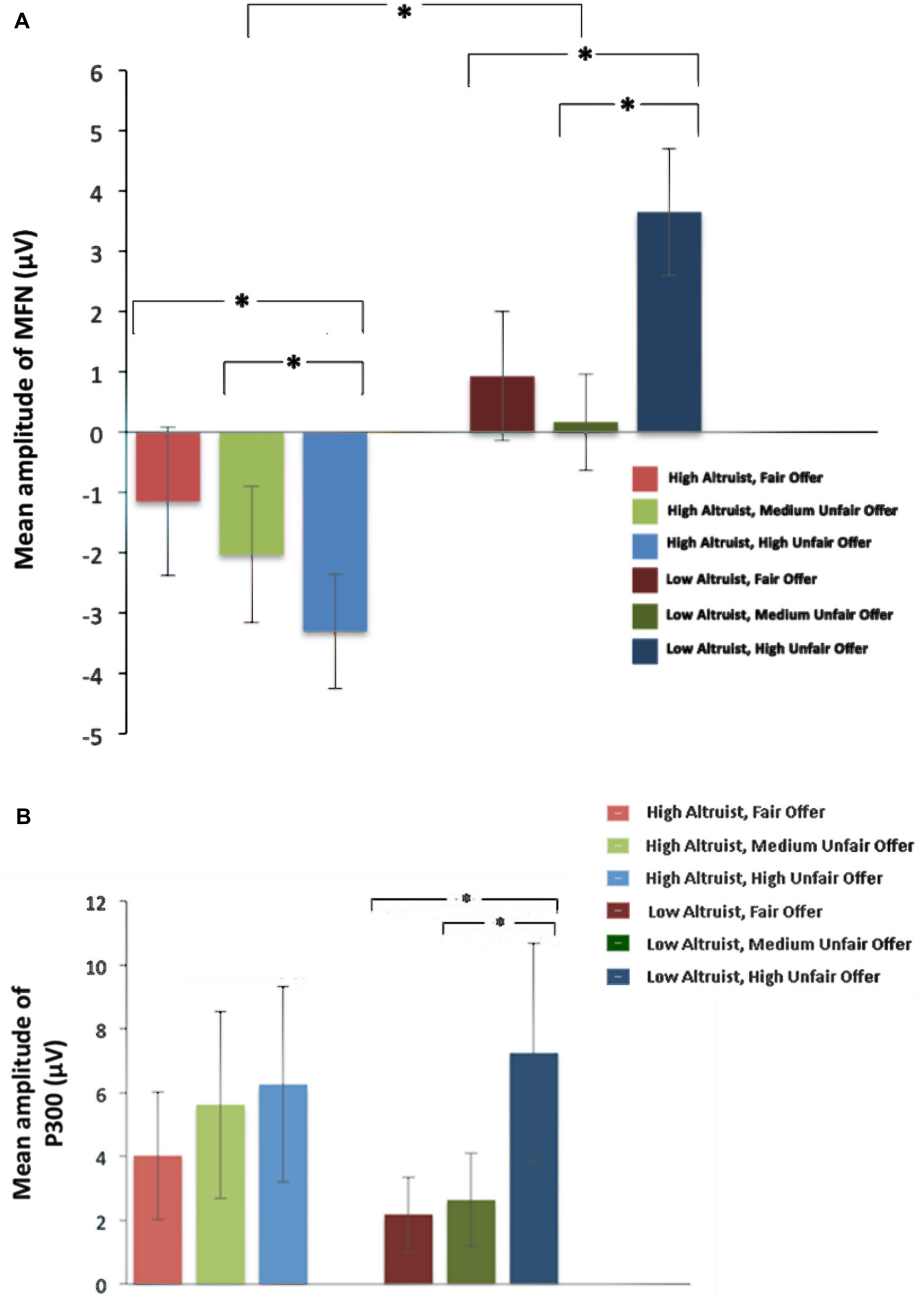
**Mean amplitude of MFN and P300 for high and low altruists at different fairness levels. (A)** MFN amplitude recorded in Fz channels. **(B)** P300 amplitude recorded in Pz channels.^∗^Significant difference refers to a *p* < 0.05. Error bars represent SE.

For the MFN amplitude, gender as a covariate, a 2 (altruistic tendency: High, Low) × 3 (fairness: Fair, Medium Unfair, High Unfair) × 3 (electrode location: Fz, FCz, Cz) rm-ANOVA revealed a significant main effect of altruistic tendency, *F*(1,27) = 5.63, *p* = 0.03: amplitude of high altruists (-1.13 ± 0.9 μV) was markedly greater than low altruists (1.75 ± 0.86 μV). The interaction between altruism level and fairness was significant, *F*(2,54) = 14.53, *p* < 0.001. More specifically, amplitudes seen in high altruists and low altruists varied in different ways across the three allocations. For high altruists, the significant difference across allocations [*F*(2,27) = 6.68, *p* = 0.004] showed that MFN amplitude of high unfair offers (-2.52 ± 0.89 μV) was more negative-going than fair offers (0.031 ± 1.03 μV) and medium unfair offers (-0.94 ± 0.9 μV), (*p* = 0.004, *p* = 0.05), whereas the medium unfair offers (-0.94 ± 0.9μV) did not differ significantly with fair offers (0.031 ± 1.03 μV), (*p* = 0.60). Low altruists also showed significant variance in fairness consideration, *F*(2,27) = 10.31, *p* < 0.001: MFN in fair offers (1.15 ± 1.03 μV) and medium unfair offers (0.60 ± 0.91 μV) was more negative-going than in high unfair offers (3.49 ± 0.90 μV), (*p* = 0.008, *p* = 0.001); but no difference was found between medium unfair and fair offers, *p* = 0.90 (**Figure 3A**). A non-significant main effect reflected no difference across fairness levels, *F*(2,54) = 1.69, *p* = 0.20. The main effect of electrode location was not significant, *F*(2,54) = 0.90, *p* = 0.37. In addition, there were no interactions between electrode location and altruistic tendency, *F*(2,54) = 3.74, *p* = 0.06 or fairness and electrode location, *F*(4,108) = 0.32, *p* = 0.80. Likewise, no significant interaction was found between electrode location, fairness and altruistic tendency, *F*(4,108) = 0.38, *p* = 0.76.

For the amplitude of P300, we also considered gender as a covariate, a 2 (altruistic tendency: High, Low) × 3 (fairness: Fair, Medium Unfair, High Unfair) × 2 (electrode location: Pz, Cz) ANOVA yielded a significant interaction between altruistic tendency and fairness level, *F*(2,54) = 5.48, *p* = 0.008. Simple effect analysis found that P300 showed a significant difference of fairness levels for low altruists, *F*(2,27) = 117.17, *p* < 0.001 but not for high altruists, *F*(2,27) = 1.50, *p* = 0.24. For low altruists, P300 amplitude for high unfair offers (6.33 ± 1.06 μV) was more positive than fair offers (1.60 ± 1.05 μV) and medium unfair offers (1.97 ± 0.80 μV), (both *p* < 0.001), but difference between fair offers (1.60 ± 1.05 μV) and medium unfair offers (1.97 ± 0.80 μV) was not significant, *p* = 0.66 (**Figure 3B**). No main effect of altruistic tendency was found, *F*(1,27) = 1.35, *p* = 0.26. The main effect of fairness did not reach significant, *F*(2,54) = 2.71, *p* = 0.08. No significant interaction was found between altruistic tendency and electrode location [*F*(1,27) = 0.008, *p* = 0.93] or between electrode location and fairness [*F*(2,54) = 0.52, *p* = 0.59]. The interaction among electrode location, fairness level and altruistic tendency was also not significant, *F*(2,54) = 0.14, *p* = 0.86.

To isolate variance in the ERP associated with the MFN and P300 from other overlapping ERP components, we created difference waves by subtracting each low altruist ERP from its appropriate corresponding high altruist ERP (Hajcak et al., 2005; Holroyd and Krigolson, 2007). Specifically, for each participant and channel, we created three difference waves by (1) subtracting the fairness ERP in the low altruist condition from the high altruist condition, creating a “fairness” difference wave; (2) subtracting the medium unfairness ERP in the low altruist condition from the high altruist condition, creating a “medium unfairness” difference wave; (3) subtracting the high unfairness ERP in the low altruist condition from the high altruist condition, creating a “high unfairness” difference wave. The amplitude of each difference wave was measured for each participant and electrode as the most negative deflection and the most positive deflection within the 0–800 ms following stimulus onset. Consistent with previous studies (Gering and Willoughby, 2002; Hajcak et al., 2005), MFN amplitude was evaluated at channel Fz, FCz, Cz, and P300 amplitude was evaluated at channel Cz, Pz, where they are normally maximal.

Following previous research, we adopted the algorithm of Holroyd and Krigolson (2007) to measure if MFN was affected by late positive component (especially P300). We carried out two sets of *t*-tests to compare results in the FCz and Pz locations, first for high altruists and second for low altruists. Results revealed that high altruistic tendency participants showed significantly larger amplitude at FCz than at Pz in all fairness conditions. For fair offers, the amplitude at FCz (-0.27 ± 3.65 μV) was significantly greater than at Pz (4.55 ± 3.00 μV), *t*(14) = -6.14, *p* < 0.001, and in medium unfair offers, amplitude in FCz (-1.25 ± 4.18 μV) was also significantly greater than in Pz (3.21 ± 3.45 μV), *t*(14) = -4.06, *p* < 0.01. Moreover, in high unfair offers, amplitude in FCz (-3.15 ± 3.44 μV) was significantly more positive than in Pz (1.59 ± 4.81 μV), *t*(14) = -3.32, *p* < 0.01. *t*-tests in the subgroup of low altruistic tendency participants showed that FCz (0.76 ± 3.91 μV) was greater than Pz for fair offers (2.61 ± 3.40 μV),*t*(14) = -2.17, *p* < 0.05. The same pattern emerged for medium unfair offers, (0.27 ± 0.75 μV; 2.55 ± 3.12 μV, *t*(14) = -2.64, *p* < 0.05), but the *t*-test result for high unfair offers found no significant difference between FCz (2.93 ± 3.77 μV) and Pz (3.84 ± 3.28 μV), *t*(14) = -1.09, *p* = 0.296. All of these results indicated that the MFN components were mainly distributed in the front of the scalp, not significantly affected by P300.

Additional evidence of the relationship between P300 and behavioral performance was obtained by testing the correlations between P300 responding to fairness levels in either high or low altruists and their behavioral performance in each group (as there is no punitive behavior confronting fair offers in either group, here we only consider medium unfair and high unfair conditions for both groups). For high altruists, behavioral performances under both medium unfair situation and high unfair situation were correlated significantly with corresponding P300s [*r*(15) = 0.57, *p* < 0.01; *r*(15) = 0.84, *p* < 0.001]. And the consistent behavioral patterns remained significant even when gender was controlled [*r*(12) = 0.86, *p* < 0.001; *r*(12) = 0.55, *p* < 0.05]. For low altruists, their behavioral performance under medium unfair and high unfair offers correlated significantly with corresponding P300 [*r*(15) = 0.61, *p* < 0.05; *r*(15) = 0.52, *p* < 0.05]. Also, this relationship remained significant when gender was controlled [*r*(12) = 0.61, *p* < 0.05; *r*(12) = 0.59, *p* < 0.05]. In other words, participants’ behavioral performance was predicted by the P300 under corresponding condition, and the relationship was not diminished when the relationship between gender and these two variables were taken into account.

## Discussion

This study tested whether altruistic tendency affects altruistic punishment and examined the neural process of fairness consideration. Consistent with prior research, third parties were more likely to punish unfair offers than fair offers, even at expense to themselves. However, altruistic tendency appeared to influence third-party punishment, in that high altruists imposed more of this type of punishment than low altruists. ERP results also indicated that altruistic tendency modulated the fairness consideration of the outcome. For high altruists, high unfair offers elicited larger MFN than medium unfair offers and fair offers; for low altruists, in contrast, fair offers elicited larger MFN, and high unfair offers caused the minimal MFN, which suggest that the altruistic tendency effect influence fairness consideration in the early stage of evaluation.

The behavioral results replicated third-party punishments and extend previous research finding altruistic tendency influences the punishments. In the experiment, all participants were paid 50 Yuan as initial endowment, and they were informed that they can spend 15 Yuan to alter the unfair allocation to the fair one; the cost could not be compensated within expectation, after the pay cost, the third party (participant) would own the least payment in three. However, most participants chose irrational altruistic punishment, thus support the inequity aversion theory. The present research extends our understanding of altruistic behaviors by focusing on individual differences in third-party punishment. Third-party punishment, as a kind of irrational behavior in economic decision making, is not shown by all people. Economic societies are constituted by members with a variety of altruistic tendencies. Therefore, further research is necessary to consider these individual differences. It will be particularly important to explore the psychological and neural mechanism of fairness consideration toward population who show less altruistic punishments.

Use of the ERP technique enabled us to explore how altruistic tendency affects fairness consideration. Specifically, for high altruists, unfair offers elicited a larger MFN and for low altruists, fair offers elicited larger MFN. According to the expectancy deviation theory of MFN, unexpected outcomes cause a larger MFN (Oliveira et al., 2007); in our study, the same allocation elicited different MFN reaction patterns, which suggests that high and low altruists hold differential expectations about allocation. Hence, MFN was enhanced when high altruists saw the unfair allocation because it conflicted with their expectations, and it was enhanced when low altruists saw the fair allocation, which conflicted with their expectations.

The ERP results shed light on the relationship between the altruistic tendency and third-party punishment, in addition to providing further electrophysiological evidence in support of the inequity aversion hypothesis. Previous studies who studied the decision-making process in the dictator game showed that decisions are the result of a two-step process. In the first step, decision makers generate an automatic, intuitive proposal. The second step is a more deliberative phase in which decision makers adjust their proposals based on motivation and cognitive resources, a process that is modulated by social context, such as the perceived interpersonal closeness of the dictator with the receiver (Cappelletti et al., 2011; Grimm and Mengel, 2011; Rand et al., 2012; Cornelissen et al., 2013). In line with the social intuitionist model, the current ERP results suggest that the effect of altruistic tendency on altruistic punishment occurs in the early stage of the outcome evaluation, which provides further cognitive neuroscience evidence for the intuition dominant two-step processing theory. An important finding in this regard is that high and low altruists appeared to differ in inequity aversion. More specifically, for high altruists, the aversion to unfairness elicited greater MFN and led to paid altruistic punishment in more than 95% of the tasks; for low altruists, their concern was more about pursuit of their maximal self-interests. From the perspective of the low altruists, the dictator should pursue the maximization of self-interest, and high unfair allocation may be an expectable result. Because unfair offers do not trigger strong aversion, unfair outcomes elicited a smaller MFN and caused less punishment.

The ERP results also showed that high altruists had more negative-going MFN in response to high unfair offers, whereas there was no significant difference between medium unfair offers and fair offers. By contrast, for low altruists, MFN was significantly larger for fair offers compared with low and high unfair offers. This shows that MFN in all allocations for high and low altruists was binary, not ternary. MFN relates to a rough primary processing for allocations, which only evaluates whether the outcome was good or not (Hajcak et al., 2006; Leng and Zhou, 2010). Although many studies have found that MFN may represent more complicated information regarding outcomes, likely to be ternary or even polynary, the presentation of outcome materials in previous research was apparent and no subsequent task was introduced after the outcome appeared (Leng and Zhou, 2010; Luo et al., 2011; Qu et al., 2013a). In our experiment, after the distribution outcome was given, participants were required to decide whether to pay to punish the dictator, so participants could only make a simple dichotomous choice. For the behavioral results, we observed that the low altruists showed less frequency of the third-party punishment behaviors toward high unfair offers. For MFN, the high altruists showed larger MFN related to high unfair offers than moderate unfair offers and fair offers, but the low altruists showed the opposite patterns. We infer that because MFN occurs in the early stage of the outcome evaluation, it just reflects the awareness of fairness rather than affect the punishment behaviors of the participants. Therefore, behavioral results were inconsistent with the MFN results.

For high altruists, there was no significant difference in P300 depending on the fairness of the allocation; for low altruists, high unfair allocation induced more positive P300. Both for high altruists and for low altruists, behavioral performances under both medium unfair situation and high unfair situation were correlated significantly with corresponding amplitude of P300. More specifically, P300 might be related to altruist’s punishments, larger amplitude of P300 predicts more third-party punishments.

This study only explored the influence of altruistic tendency on fairness consideration, and did not explore how altruistic tendency impacts the participants’ willingness to pay to punish after becoming aware of the unfairness. In the end of the experiment, many participants reported that they had been aware of the unfairness in the tasks. However, they were not willing to pay such a high price to change the unfair allocation. Thus, further research will examine the trade-off processing in deciding the costly punishment.

## Conclusion

This study is the first to examine the influence of altruistic tendency on third-party punishment and the neurophysiological process underlying fairness consideration. Behavioral results showed that compared with low altruists, high altruists delved out more third-party punishment; ERP results indicated that altruistic tendency also affected the awareness of fairness in the early stage of evaluation: for high altruists, high unfair allocation elicited more negative-going MFN amplitude, but for low altruists, fair offer triggered greater MFN. Specifically, these results demonstrate a stronger inequity aversion in high altruists than in low altruists, which may cause more punishment behavior.

## Conflict of Interest Statement

The authors declare that the research was conducted in the absence of any commercial or financial relationships that could be construed as a potential conflict of interest.
